# Characterization and Stage-Dependent Lineage Analysis of Intermediate Progenitors of Cortical GABAergic Interneurons

**DOI:** 10.3389/fnins.2021.607908

**Published:** 2021-07-08

**Authors:** Shigeyuki Esumi, Makoto Nasu, Takeshi Kawauchi, Koichiro Miike, Kento Morooka, Yuchio Yanagawa, Tatsunori Seki, Kenji Sakimura, Takaichi Fukuda, Nobuaki Tamamaki

**Affiliations:** ^1^Department of Anatomy and Neurobiology, Graduate School of Medical Sciences, Kumamoto University, Kumamoto, Japan; ^2^Department of Health Sciences, Faculty of Life Sciences, Kumamoto University, Kumamoto, Japan; ^3^Laboratory of Molecular Life Science, Institute of Biomedical Research and Innovation, Foundation for Biomedical Research and Innovation at Kobe (FBRI), Kobe, Japan; ^4^Institute of Molecular Embryology and Genetics, Kumamoto University, Kumamoto, Japan; ^5^Kumamoto University Hospital, Kumamoto, Japan; ^6^Department of Genetic and Behavioral Neuroscience, Gunma University Graduate School of Medicine, Maebashi, Japan; ^7^Department of Histology and Neuroanatomy, Tokyo Medical University, Tokyo, Japan; ^8^Department of Cellular Neurobiology, Brain Research Institute, Niigata University, Niigata, Japan; ^9^Department of Morphological Neural Science, Graduate School of Life Sciences, Kumamoto University, Kumamoto, Japan

**Keywords:** GABAergic neuron progenitors, lineage, cortical development, fate analysis, laminar distribution

## Abstract

Intermediate progenitors of both excitatory and inhibitory neurons, which can replenish neurons in the adult brain, were recently identified. However, the generation of intermediate progenitors of GABAergic inhibitory neurons (IPGNs) has not been studied in detail. Here, we characterized the spatiotemporal distribution of IPGNs in mouse cerebral cortex. IPGNs generated neurons during both embryonic and postnatal stages, but the embryonic IPGNs were more proliferative. Our lineage tracing analyses showed that the embryonically proliferating IPGNs tended to localize to the superficial layers rather than the deep cortical layers at 3 weeks after birth. We also found that embryonic IPGNs derived from the medial and caudal ganglionic eminence (CGE) but more than half of the embryonic IPGNs were derived from the CGE and broadly distributed in the cerebral cortex. Taken together, our data indicate that the broadly located IPGNs during embryonic and postnatal stages exhibit a different proliferative property and layer distribution.

## Introduction

Brain function relies on the concordance between excitatory and inhibitory neuron activities, and excitatory–inhibitory imbalances are associated with many psychiatric disorders (Bozzi et al., 2012; Marín, 2012; Lim et al., 2018; Sohal and Rubenstein, 2019). Excitatory and inhibitory neurons are generated in different brain regions. In the developing cerebral cortex, glutamatergic excitatory neurons originate from the dorsal ventricular zone (VZ) of the cerebral cortex, whereas GABAergic inhibitory neurons (IPGNs) arise from the medial ganglionic eminence (MGE), caudal ganglionic eminence (CGE), or preoptic area (POA) and migrate tangentially to the cerebral cortex (Anderson et al., 1997, Anderson et al., 2001; Tamamaki et al., 1997, 2001; Lavdas et al., 1999; Wichterle et al., 1999; Miyata et al., 2001; Noctor et al., 2001; Nery et al., 2002). GABAergic neuron progenitors change from tangential to radial migration after they enter the pallium and move to their final location (Tanaka et al., 2006, 2009; Miyoshi and Fishell, 2011; Bartolini et al., 2013). A majority of cortical GABAergic neuron progenitors in the MGE and POA express Nkx2-1 (Xu et al., 2004; Butt et al., 2005; Wonders and Anderson, 2006; Lim et al., 2018). Fate mapping analyses have shown that the MGE- and POA-derived progenitors are distributed to broad areas of the forebrain (Harwell et al., 2015; García et al., 2016; Mayer et al., 2016; Sultan et al., 2016). Meanwhile, approximately 30% of all cortical GABAergic neuron progenitors are derived from the CGE, and they are preferentially distributed to the superficial layers (Miyoshi et al., 2010; Miyoshi and Fishell, 2011).

Neural progenitors (also referred to as apical progenitors or radial glia) are located along the ventricles, and apical progenitors of the excitatory neurons produce intermediate progenitors, which divide at the subventricular zone (SVZ) of the dorsal telencephalon to generate two neurons (Noctor et al., 2004; Wu et al., 2005; Pilz et al., 2013). Recently, it was reported that apical progenitors of the inhibitory neurons also produce intermediate progenitors (Wu et al., 2011; Hansen et al., 2013; Petros et al., 2015). Lineage tracing studies have revealed that the Nkx2-1-lineage progenitor cells undergo symmetric and asymmetric division in the SVZ of the MGE (Brown et al., 2011; Ciceri et al., 2013; Sultan et al., 2014). However, the intermediate progenitors of IPGNs have not yet been well characterized. In primates, including humans, many IPGNs are observed at the cortical SVZ as well as the SVZ of the ganglionic eminence at embryonic stages (Letinic et al., 2002; Zecevic et al., 2005, 2011; Petanjek et al., 2009; Jakovcevski et al., 2011; Yu and Zecevic, 2011), suggesting broad expansion of IPGNs in primates. These IPGNs in the cortical SVZ derive mainly from the outer SVZ of the ganglionic eminence (Hansen et al., 2013; Ma et al., 2013). The CGE, in particular, contributes to a large number of the cortical GABAergic neurons in humans (Hansen et al., 2013).

By contrast, we and others have observed a small number of IPGNs in the cortical-SVZ in embryonic and postnatal mouse brains (Inta et al., 2008; Wu et al., 2011). These IPGNs express neuronal and proliferative markers at the perinatal stage in glutamic acid decarboxylase 67 (GAD67)-GFP knock-in mice, which are widely used to visualize GABAergic neurons and IPGNs. Our previous study revealed that ∼1.5% of the perinatal cortical GAD67-GFP-positive cells self-renew and produce a small number of cortical GABAergic neurons (Wu et al., 2011). Furthermore, a small number of cortical GABAergic neurons are generated in the cortical-SVZ (Inta et al., 2008) and early postnatal dorsal white matter (Riccio et al., 2012). Thus, unlike the intermediate progenitors of the excitatory neurons, IPGNs are widely dispersed from the subpallium to the cerebral cortex. However, the distribution patterns of IPGNs and their fates in adult cerebral cortex are poorly understood.

In this study, we performed systematic analyses of spatiotemporal distribution patterns of IPGNs in mice. Our lineage tracing analyses indicated that the laminar distributions of the embryonic and postnatal IPGNs differ. Late embryonic IPGNs tended to differentiate into reelin-positive (Reln^+^) or vasoactive intestinal peptide positive (Vip^+^) GABAergic interneurons but were also able to differentiate into parvalbumin-positive (Pvalb^+^) or somatostatin-positive (Sst^+^) neurons, suggesting that the IPGNs observed at the late embryonic stage are mainly derived from the CGE, rather than the MGE. Consistently, IPGNs were found primarily in the CGE-SVZ but also the MGE-SVZ and, to lesser extent, in the mantle zone of the ganglionic eminence and cortical-SVZ/intermediate zone (IZ) at embryonic stages. These findings indicate that IPGNs exhibit a unique spatiotemporal distribution and significantly contribute to several subtypes of GABAergic interneurons in adult cerebral cortex.

## Materials and Methods

### Production of GAD67-CrePR Knock-in Mouse

Progesterone inducible Cre recombinase (CrePR) was generated to fuse CrePR and progesterone receptor ligand binding motif genes (Kellendonk et al., 1996; Kitayama et al., 2001). To produce GAD67-knock-in CrePR mice, we designed a targeting vector in which a CrePR recombinase gene was inserted into the translational initiation site of the *GAD67* gene in frame (Supplementary Figure 1). A knock-in vector pGAD67CrePRTV contained a 3 kb fragment at the 5′ side, a CrePR gene placed behind the GAD67 translational start, a *Pgk*-neo-p(A) cassette flanked by two Flp recognition target (frt) sites, a 7 kb fragment at the 3′ side, and an MC1 promoter-driven diphtheria toxin gene. Culture of embryonic stem (ES) cells and generation of chimeric mice were performed as described previously (Kitayama et al., 2001; Higo et al., 2009). Briefly, a linearized pGAD67CrePRTV was introduced into C57BL/6 mouse ES cells (RENKA) and then, G418-resistant clones were picked up. Homologous recombined ES clone was identified by Southern blotting. To produce germline chimera, the selected ES cells were microinjected into eight cell-stage embryos of the CD-1 mouse strain. The germline chimeras of GAD67-CrePR mice were crossed with C57BL/6 mice to generate the GAD67-CrePR mouse line. Because the knock-out of both GAD67 alleles is lethal at birth (Asada et al., 1997), mice heterozygous for the altered GAD67 allele were used for all the observations in this study. Genotypes were identified by Southern blot hybridization or PCR. Tail genomic DNA was digested with Spe-I or Afl-II and hybridized with a 5′ probe or a 3′ probe, respectively. PCR was performed with specific 3′. The sequence of each primer and the approximate length of the amplified DNA fragments are described as follows: Gad1CrePR, g67-2 (5′-TTCCGGAGGTACCACACCTT-3′), g67-5 (5′-TAAGTCGACGCTAGCGAGCGCCTCCCCA-3′), and CreR1 (5′-TTGCCCCTGTTTCACTATCC-3′); wild type, 1.8 kbp; mutant: 1.4 kbp.

### Animals

Mice were housed and treated in strict accordance with the rules for animal care and use for research and education of Kumamoto University. They were kept in cages and exposed to a 12 h light/dark cycle with food and water provided *ad libitum*. GAD67-CrePR mice express a mifepristone-inducible CrePR under the control of the GAD67 gene. To label GAD67^+^ cells, 0.05 mg/g body weight of mifepristone (Sigma-Aldrich, St. Louis, MO, United States) was administered to pregnant GAD67-CrePR mice, or 0.05 mg/g body weight mifepristone was administered to postnatal day 0 pups by intraperitoneal (i.p.) injection. Mifepristone was dissolved in 100% ethanol to 50 mg/mL and then diluted in corn oil (Wako, Japan) to 5 mg/mL. To prevent abortions in the pregnant mice, progesterone dissolved in corn oil (20 mg/mL, stored at 4°C; Sigma-Aldrich, St. Louis, MO, United States) was administered at 0.15 mg/g body weight once a day between E12 and E18 (Rose et al., 2009). To detect Cre-mediated recombination in GAD67-CrePR knock-in mice, mice that express TdTomato reporter upon Cre-mediated recombination were used (Jackson stock no. 007909 with an ICR background) (Madisen et al., 2010). GAD67-CrePR knock-in male mice were mated with R26-TdTomato (Ai9) reporter female mice or ICR mice (Oriental Yeast, Japan). As a previous study showed that CreER-mediated reporter recombination is initiated 6 h after tamoxifen administration, intermediate progenitors of IPGNs and GABAergic neurons are sufficiently labeled with TdTomato 24 h after the injection (Zervas et al., 2004). RFP antibody were utilized for enhanced visualization of TdTomato expression. Newborn pups were allowed to develop up to 3 weeks. Pregnant mice and juvenile mice (3 weeks) were anesthetized as described below and perfused with a fixative for immunohistochemistry.

### Fixation

The mice were anesthetized with sodium pentobarbital (50 mg/kg body weight, i.p.) and perfused transcardially with 10 mL PBS [0.9% (w/v) saline buffered with 5 mM sodium phosphate, pH 7.4], followed by 50 mL PBS containing 4% (w/v) formaldehyde. The brains were removed, postfixed with the same fixative for 12 h, and then cryoprotected in 25% sucrose in PBS overnight. The brains were sliced into 50 μm-thick coronal sections on a cryostat.

### Immunohistochemistry

Immunohistochemistry was performed on the brains of male and/or female mice. Immunohistochemistry was performed as described previously (Tamamaki et al., 2003; Miyamoto and Fukuda, 2015). Briefly, brain sections were rinsed in PBS several times and incubated overnight at room temperature with primary antibody diluted in incubation buffer (0.3% Triton X-100, 5% donkey serum, and 0.01% sodium azide in PBS). The primary antibodies used in this experiment are listed in Table 1. Secondary antibodies were goat anti-rabbit or anti-rat IgG conjugated with Alexa Fluor 488 and donkey anti-rabbit IgG conjugated with Alexa Fluor 594 (1:500; Molecular Probes, Eugene, OR, United States), or goat anti-mouse IgM conjugated with Alexa Fluor 594 (1:500; Chemicon, Temecula, CA, United States). The sections were mounted onto MAS-coated glass slides after staining.

**TABLE 1:** Primary antibodies used in this study.

**Antibody**	**Dilution**	**Source or reference**	**Animal**
Anti-BrdU	1:200	Abcam	Rat
Anti-GABA	1:5,000	Sigma	Rabbit
Anti-GFP	1:500	Nacalai Tesque	Rat
Anti-Ki67	1:500	Epitomics	Rabbit
Anti-NeuN	1:1,000	Chemicon	Mouse
Anti-nestin	1:200	BD Bioscience	Mouse
Anti-parvalbumin	1:2,000	Sigma	Mouse
Anti-PCNA	1:50	Novocastra	Mouse
Anti-pHH3	1:200	Millipore	Rabbit
Anti-somatostatin	1:200	Millipore	Rabbit
Anti-Tuj1	1:500	Covance	Mouse
Anti-RFP	1:500	Tomioka et al., 2015	Rabbit
Anti-Vip	1:500	ImmunoStar	Rabbit

### EdU Labeling

To detect proliferative IPGNs, EdU, an analog of the nucleoside thymidine that incorporates into replicating DNA was injected to the GAD67-CrePR;Ai9 mice 24 h after the mifepristone administration. This inducible genetic fate mapping method enables labeling of postnatally proliferative GAD67^+^ IPGNs and tracing of their lineage in the mature brain. Direct labeling of EdU was performed after incubations with primary antibody. Tissue sections were incubated with 1% bovine serum albumin in PBS for 1 h and then with a freshly prepared cocktail containing 50 mM *Tris* (pH 7.4), 150 mM NaCl_2_, 2 mM CuSO_4_, 10 μM Alexa Fluor 647-azide (from 1 mM stock in dimethyl sulfoxide, A10277; Thermo Fisher, Waltham, MA, United States), and 10 mM sodium ascorbate (added last from 0.1 M stock) in the dark for 2 h. Sections were washed in PBS three times (protocol modified from that described by Hua and Kearsey (2011)]. Then, sections were incubated with secondary antibodies.

### Quantifications and Statistical Analysis

EdU-labeled and immunoreactive cells were counted under a confocal microscope (Nikon C2) individually by capturing three-dimensional images at a 1–2 μm thickness. Total thickness of three-dimensional images in the depth direction were more than 15 μm. More than 8 hemisphere of cortical slices and more than 50 cells were analyzed from a brain in each experiment, respectively. Data are presented as means ± standard errors (SEs). Statistical significance was determined by two-sided unpaired Student’s *t*-tests, with a *P*-value of <0.05 considered statistically significant.

## Results

### A Small Proportion of GAD67^+^ Intermediate Progenitors of GABAergic Inhibitory Neurons (IPGNs) Proliferate in Perinatal Cerebral Cortex

**FIGURE 1:**
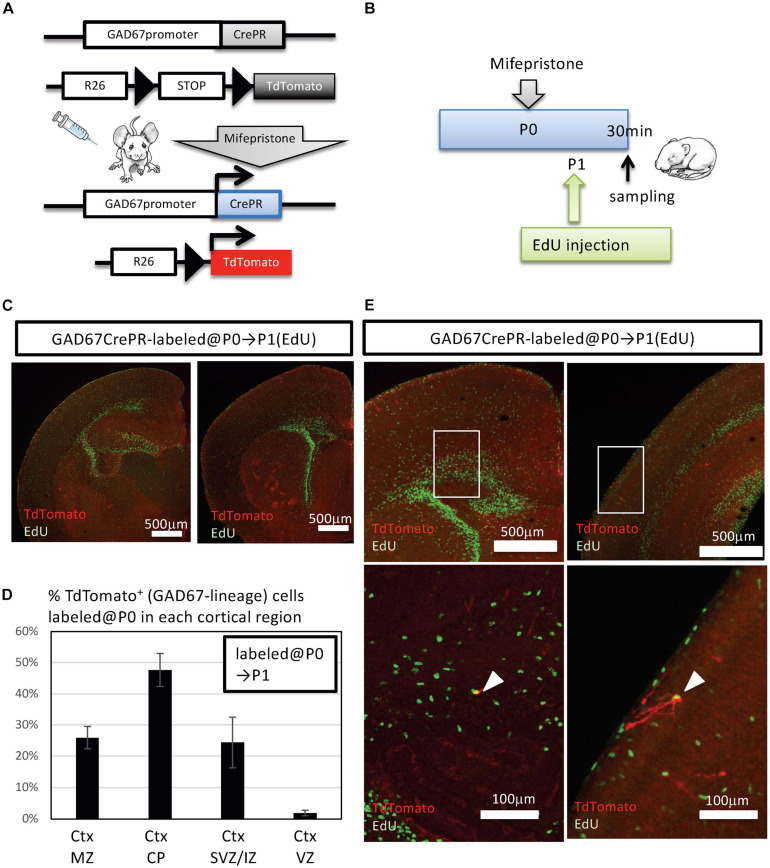
Fate mapping analysis of postnatal GAD67^+^ intermediate progenitors of GABAergic inhibitory neurons (IPGNs) using GAD67-CrePR mouse. **(A)** Strategy for labeling GAD67-lineage cells by mifepristone-dependent recombination. Cre recombinase is restricted to GAD67-expressing cells after mifepristone treatment. The Rosa26 (R26)-floxed-TdTomato (Ai9) allele expresses TdTomato following Cre-mediated recombination. **(B)** Experimental design for labeling GAD67-lineage cells at the perinatal stage. Mifepristone was injected i.p. into GAD67-CrePR;Ai9 mice at P0, and EdU was injected at P1. Mifepristone- and EdU-treated mice were analyzed 30 min after EdU injection. **(C)** Immunohistochemistry for TdTomato (red) expression and EdU (green) at P1. An anti-RFP antibody was utilized to enhance TdTomato fluorescence. **(D)** Percentages of TdTomato^+^ GAD67-lineage cells labeled at P0 in each cortical layer. Ctx, cortex; MZ, marginal zone; CP, cortical plate; SVZ/IZ, subventricular zone/intermediate zone. Error bars represent SDs (*n* = 3 brains). **(E)** Representative images of TdTomato^+^ GAD67-lineage cells (red) with EdU (green) in perinatal subventricular zone/intermediate zone (left) and marginal zone (right). Lower images show higher magnification of regions outlined with white boxes. White arrowheads indicate the double-positive cells.

We previously reported the presence of a small number of IPGNs in the SVZ and even in the IZ of the cerebral cortices of GAD67-GFP knock-in mice at the perinatal stage (Wu et al., 2011). However, the number of postnatally generated IPGNs in the cortical-SVZ (or cortical-IZ) in rodents is much lower than that in primates, and it is not known whether IPGNs survive and mature in adult rodent cortex. To assess the distribution of cortical GABAergic neurons generated postnatally from IPGNs, we utilized GAD67-CrePR mice, which expressed CrePR, a mifepristone-inducible Cre, in GABAergic neurons and IPGNs (Figure 1A). To detect the initial population of postnatally proliferating IPGNs in the cortex, we performed immunohistochemistry using GAD67-CrePR;Rosa26-floxed-TdTomato (Ai9 line) mice at postnatal day 1 (P1) that had been administered mifepristone at P0 and EdU 30 min before fixation (Figure 1B). GAD67^+^ TdTomato^+^ cells were broadly distributed throughout the brain, including the cerebral cortex. Although EdU^+^ cells were mainly located in the neurogenic niches, they could be found throughout the brain, including the superficial layers of the cortex (Figures 1C–E).

A very small number of GAD67^+^ TdTomato^+^ cells colabeled with EdU, and these double-positive cells were observed in the cortical SVZ/IZ and cortical marginal zone (Figure 1E). At P1, 0.4 ± 0.4% (3/615 cells from three brains) of the GAD67^+^ TdTomato^+^ cells were EdU^+^, consistent with our previous analyses showing GAD67^+^ perinatal IPGNs in the cortex (Wu et al., 2011). Therefore, we defined GAD67^+^ TdTomato^+^ EdU^+^ double-positive cells as IPGNs. Thus, approximately <0.5% of labeled cells were postnatally proliferative IPGNs in mouse cortex at P1.

### A Small Proportion of GAD67^+^ Intermediate Progenitors of GABAergic Inhibitory Neurons (IPGNs) Proliferate and Survive in Cerebral Cortex During Perinatal Development

**FIGURE 2:**
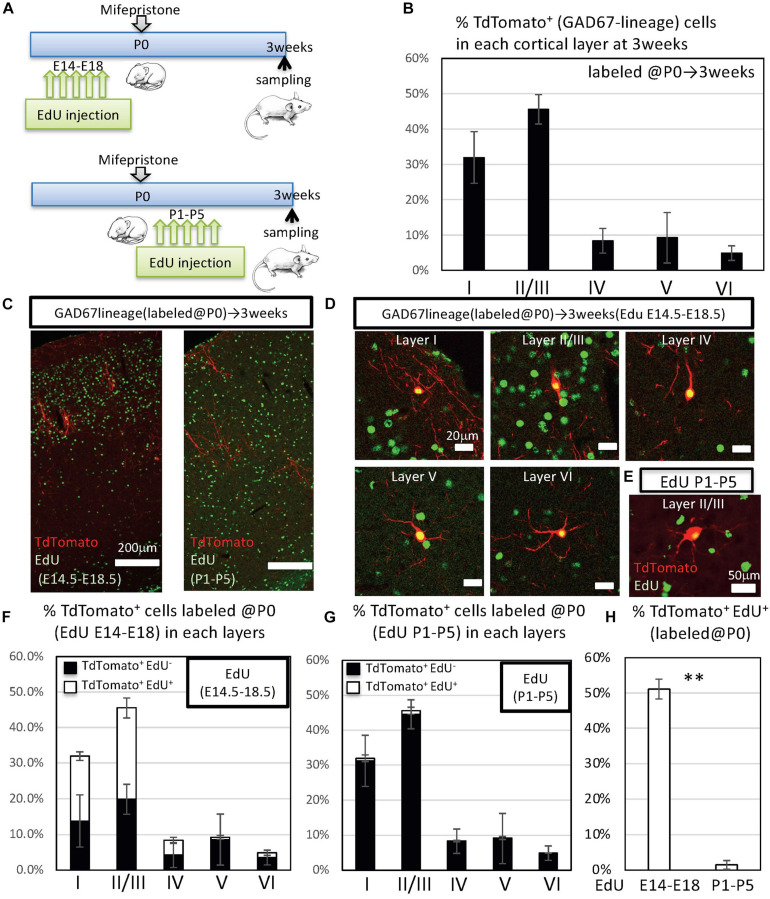
Fate mapping analysis of GAD67^+^ IPGNs using GAD67-CrePR mouse labeled at P0. **(A)** Experimental design for labeling IPGNs at the perinatal stage. Mifepristone was administered to pregnant GAD67-CrePR;Ai9 mice at P0, and EdU was injected i.p. from E14.5 to E18.5 or P1 to P5. Mifepristone- and EdU-treated mice were analyzed at 3 weeks of age. IPGNs, intermediate GABAergic neuron progenitors. **(B)** Percentages of TdTomato^+^ GAD67-lineage cells in each cortical layer labeled at P0. Error bars represent SDs (*n* = 9 brains). **(C)** Coronal section of GAD67-CrePR;Ai9 mice brain at 3 weeks with EdU staining (green). EdU was injected E14.5–E18.5 (left) P1–P5 (right) by dual-fluorescence immunohistochemistry. Immunocytochemical analyses were performed with confocal laser microscopy. **(D)** Representative images of TdTomato^+^ GAD67-lineage cells labeled at P0 (red) with EdU (E14–18) (green) in each cortical layer at 3 weeks of age. **(E)** Representative image of TdTomato^+^ GAD67-lineage cells labeled at P0 (red) with EdU (P1–P5) (green) in cortical layer II/III at 3 weeks of age. **(F)** Percentages of TdTomato^+^ cells (GAD67 lineage labeled at P0) labeled with EdU (E14–E18) in each cortical layer. Error bars represent SDs (*n* = 3 brains). **(G)** Percentages of TdTomato^+^ cells (GAD67 lineage labeled at P0) labeled with EdU (P1–P5) in each cortical layer. Error bars represent SDs (*n* = 6 brains). **(H)** Percentages of TdTomato^+^ EdU^+^ GAD67-lineage cells labeled at P0 and EdU injected from E14.5 to E18.5 (*n* = 3 brains) or P1 to P5 (*n* = 6 brains). ^∗∗^*P* < 0.01. Error bars represent SDs.

To compare the fates of proliferative GABAergic neuron progenitors between late embryonic stage and the perinatal stage, we performed a fate mapping analysis. To detect the proliferative GABAergic neuron progenitors during late embryonic stages, EdU was injected into GAD67-CrePR;Ai9 mice from E14.5 to E18.5 (late embryonic stage) or from P1 to P5 (perinatal stage) with mifepristone administered at P0 to label embryonically or postnatally proliferative IPGNs, respectively (Figure 2A). At 3 weeks after birth, TdTomato^+^ cells were broadly distributed throughout the brain, including the cerebral cortex (Figures 2B,C), hippocampus, striatum, thalamus, hypothalamus, midbrain, and cerebellar cortex (Supplementary Figure 2). TdTomato^+^ cells were observed in each cortical layer but tended to distribute in the superficial layers (i.e., layers I and II/III) (Figures 2B–D).

To further characterize these EdU^+^ and TdTomato^+^ double-positive GAD67-lineage cells in each layer, we performed immunohistochemical analyses with EdU. When labeled from P1 to P5, EdU^+^ cells were broadly distributed throughout the cortex. By contrast, when labeled at the late embryonic stages (from E14.5 to E18.5), EdU^+^ cells tended to populate the superficial layers (i.e., layers I and II/III) (Figure 2C). Approximately 51% [51.2 ± 2.8% (total 336/657 cells from three brains)] of the TdTomato^+^ GAD67-lineage cells (labeled at P0) were colabeled with EdU that was injected from E14.5 to E18.5 (Figures 2F–H). By contrast, approximately 1.5% [1.5 ± 1.2% (13/1,046 cells from six brains)] of the TdTomato^+^ GAD67-lineage cells (labeled at P0) were colabeled with EdU that was injected at P1–P5 (Figures 2F–H and Table 2). These results demonstrate that a very small number of postnatal proliferative IPGNs were represented in perinatal cortex by our fate mapping study. Thus, the proliferative rates of the GABAergic neuron progenitors during late embryonic stage were nearly 34-fold higher than that of the postnatally proliferative IPGNs (labeled at P1–P5), indicating that significantly more GABAergic neurons are produced during embryonic stages. Moreover, these GAD67^+^ embryonic proliferating cells (EdU labeled at E14.5–E18.5) were more abundant in the superficial cortical layers (i.e., layers II/III) than in the deep layers (Figures 2F,G and Table 2). These results delineate the fate of later-born embryonic GABAergic neurons and postnatally proliferative IPGNs.

**TABLE 2:** Localization of GAD67-lineage and EdU^+^ cells in 3 weeks mice treated with mifepristone at P0 and E13.5.

**Mifepristone**	**EdU**	**No. or % RFP^+^ cells in cortical layer**					
		**I**	**II/III**	**IV**	**V**	**VI**	**Total**
**No. RFP^+^ (RFP^+^ EdU^+^)**							
E13.5 (*n* = 6 brains)	E14–E18	219 (86)	291 (133)	122 (20)	309 (35)	111 (12)	1,052 (286)
E13.5 (*n* = 3 brains)	P1–P5	115 (2)	139 (3)	75 (0)	91 (1)	60 (0)	480 (6)
P0 (*n* = 6 brains)	E14–E18	240 (118)	283 (167)	74 (27)	31 (4)	22 (9)	650 (325)
P0 (*n* = 3 brains)	P1–P5	294 (5)	438 (7)	60 (1)	80 (1)	48 (0)	920 (14)
**% RFP^+^**							
E13.5 (*n* = 9 brains)		21.9 ± 5.4%	28.2 ± 5.0%	12.7 ± 4.9%	25.9 ± 8.5%	11.2 ± 3.3%	
P0 (*n* = 9 brains)		31.9 ± 7.3%	45.6 ± 4.2%	8.4 ± 3.5%	9.2 ± 7.2%	4.9 ± 2.1%	
**% RFP^+^ EdU^+^**							
E13.5 (*n* = 6 brains)	E14–E18	8.3 ± 2.4%	13.2 ± 4.9%	1.9 ± 1.1%	3.4 ± 3.5%	1.1 ± 1.1%	27.8 ± 7.5%
E13.5 (*n* = 3 brains)	P1–P5	0.4 ± 0.6%	0.6 ± 0.7%	0%	0.2 ± 0.3%	0%	1.2 ± 1.1%
P0 (*n* = 3 brains)	E14–E18	18.2 ± 1.2%	25.7 ± 2.8%	4.1 ± 0.9%	0.6 ± 0.5%	1.4 ± 0.8%	50.0 ± 2.1%
P0 (*n* = 6 brains)	P1–P5	0.8 ± 1.0%	1.1 ± 1.0%	0.1 ± 0.2%	0.2 ± 0.4%	0%	2.1 ± 1.8%

### Subtype Specification of Proliferative GAD67-Lineage Cells Derived From Postnatal GAD67^+^ Intermediate Progenitors of GABAergic Inhibitory Neurons (IPGNs)

**FIGURE 3:**
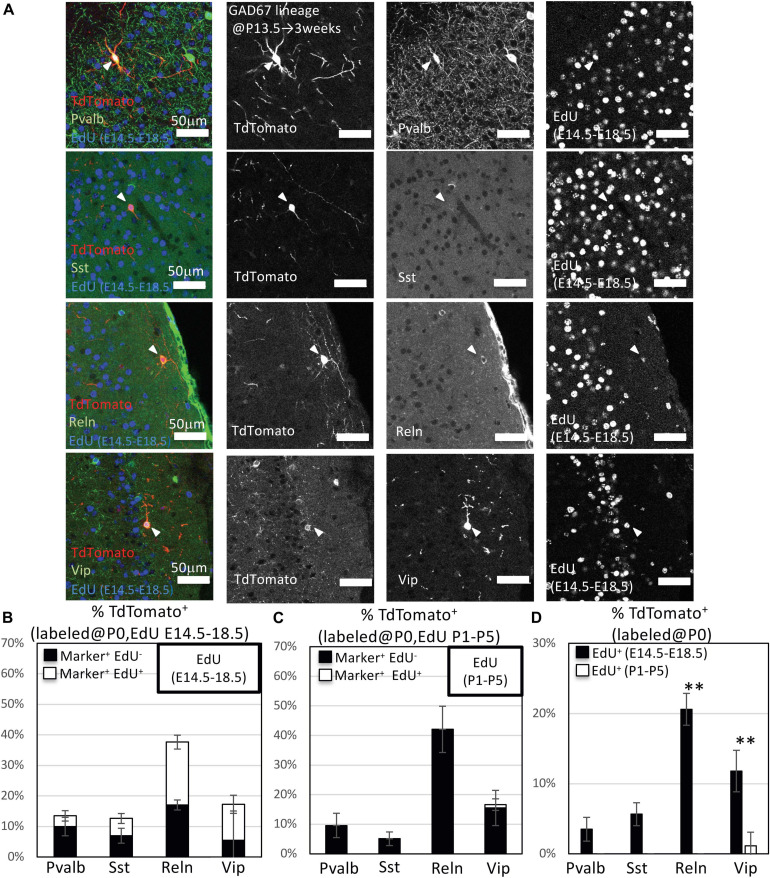
Immunohistochemistry analysis of GAD67-lineage cells labeled at P0. **(A)** Triple-fluorescence immunohistochemistry of GAD67-lineage cells (red; labeled at P0) for EdU (blue; labeled E14.5–E18.5) and GABAergic (Pvalb, Sst, Reelin and Vip) neuron markers (green) from GAD67-CrePR;Ai9 mice at 3 weeks. White arrowheads indicate the triple-positive cells. Pvalb, parvalbumin; Sst, somatostatin; Reln, reelin; Vip, vasoactive intestinal peptide. **(B)** Percentages of TdTomato^+^ cells labeled with EdU (E14–E18), and/or GABAergic (Pvalb, Sst, Reln, and VIP) neuron subtype markers. Black bars indicate the percentages of TdTomato^+^ and GABAergic neuron subtype marker double-positive cells. White bars indicate the percentages of TdTomato^+^ GABAergic neuron subtype marker and EdU^+^ triple-positive cells. Error bars represent SDs (*n* = 3 brains). **(C)** Percentages of TdTomato^+^ cells labeled with EdU (P1–P5), and/or GABAergic (Parv, Sst, Reln, and Vip) neuron subtype markers. Black bars indicate the percentages of TdTomato^+^ and GABAergic neuron subtype marker double-positive cells. White bars indicate the percentages of TdTomato^+^ GABAergic neuron subtype marker and EdU^+^ triple-positive cells. Error bars represent SDs (*n* = 3 brains). **(D)** Percentages of TdTomato^+^ EdU^+^ GABAergic neuron subtype marker^+^ GAD67-lineage cells labeled at P0 and EdU injected from E14.5 to E18.5 or P1 to P5. ^∗∗^*P* < 0.01. Error bars represent SDs (*n* = 3 brains).

To investigate subtypes of GAD67-lineage cortical GABAergic neurons that were produced during late embryonic stages (EdU administration at E14.5–E18.5) or perinatal stages (EdU administration at P1–P5), we performed immunohistochemistry with subtype markers for Pvalb^+^, Sst^+^, Reln^+^, and Vip^+^ interneurons in conjunction with EdU staining in the GAD67-CrePR;Ai9 mice administered mifepristone at P0 to label GAD67^+^ cells (Figure 3A). Among the TdTomato^+^ GAD67-lineage cells, 11.5 ± 3.8% were Pvalb^+^ (38/329 cells from six brains), 8.9 ± 4.6% were Sst^+^ (29/341 cells from six brains), 39.7 ± 5.6% were Reln^+^ (126/313 cells from six brains), and 16.6 ± 7.0% were Vip^+^ (56/338 cells from six brains) (Figure 3B and Table 3), suggesting that more than 40% of the GAD67-lineage cells labeled at P0 were Reln^+^ or Vip^+^ GABAergic neurons. These results indicate that our fate mapping strategy tends to label later-born CGE-derived GABAergic neurons rather than MGE-derived neurons. Next, we estimated the ratio of the GAD67-lineage cortical GABAergic neurons that were produced during late embryonic stages (EdU labeling at E14.5–E18.5) in each subtype. Among the TdTomato^+^ GAD67-lineage cells that were labeled with EdU at E14.5–E18.5, 3.5 ± 1.7% (6/172 cells from three brains) were double positive for Pvalb and EdU, 5.6 ± 1.6% (9/158 cells from three brains) were double positive for Sst and EdU, 20.6 ± 2.3% (32/155 cells from three brains) were double positive for Reln and EdU, and 11.8 ± 3.0% (20/172 cells from three brains) were double positive for Vip and EdU (Figure 3D). These double-positive cells were produced during late embryonic stages and later differentiated into GAD67^+^ GABAergic neurons at P0. We next investigated the ratio of postnatally proliferative GAD67^+^ IPGNs (EdU labeling at P1–P5). However, no double-positive cells were found for Pvalb (total 0/157 cells from three brains), Sst (0/183 cells from three brains), or Reln (0/164 cells from three brains) among the TdTomato^+^ GAD67-lineage cells that were labeled with EdU injected at the perinatal stage (P1–P5), whereas only 1.6 ± 1.4% (2/166 cells from three brains) were double positive for Vip and EdU (Figure 3C). The proportion of cortical GABAergic neurons produced during the late embryonic stage (E14.5–E18.5) was significantly higher than that during the perinatal stage (*P* < 0.01) for Reln^+^ and Vip^+^ GABAergic neuron subtypes. Interestingly, a small number of GAD67^+^ postnatally proliferative cells colabeled with Vip, a marker for CGE-derived GABAergic neurons (Figure 3D).

**TABLE 3:** Percentages of TdTomato^+^ cells labeled with EdU and/or GABAergic neuron markers.

**Mifepristone**	**No. or % RFP^+^ cells in cortex**				
	**EdU**	**Pvalb**	**Sst**	**Reln**	**Vip**
**Total no. RFP^+^ (Marker^+^ RFP^+^)**					
E13.5 (*n* = 6 brains)		312 (71)	315 (50)	316 (135)	321 (34)
P0 (*n* = 6 brains)		329 (38)	341 (29)	313 (126)	338 (56)
**% Marker^+^ RFP^+^**					
E13.5 (*n* = 6 brains)		22.7 ± 4.3%	15.9 ± 8.7%	42.6 ± 8.9%	10.4 ± 8.6%
P0 (*n* = 6 brains)		11.5 ± 3.8%	8.9 ± 4.6%	39.7 ± 5.6%	16.6 ± 7.0%
**Total No. RFP^+^ (Marker^+^ RFP^+^ [Marker^+^ RFP^+^ EdU^+^])**					
E13.5 (*n* = 3 brains)	E14–E18	153 (37 [5])	153 (25 [1])	156 (57 [17])	156 (6 [4])
E13.5 (*n* = 3 brains)	P1–P5	159 (34 [0])	156 (25 [0])	160 (78 [3])	156 (28 [0])
P0 (*n* = 3 brains)	E14–E18	172 (23 [6])	158 (20 [9])	155 (58 [32])	172 (29 [20])
P0 (n = 3 brains)	P1–P5	157 (15 [0])	183 (9 [0])	158 (68 [0])	166 (27 [2])
**% Marker^+^ RFP^+^ EdU^+^**					
E13.5 (*n* = 3 brains)	E14–E18	3.2 ± 4.0%	0.6 ± 1.1%	10.8 ± 2.6%	2.6 ± 1.2%
E13.5 (*n* = 3 brains)	P1–P5	0%	0%	1.8 ± 0.4%	0%
P0 (*n* = 3 brains)	E14–E18	3.5 ± 1.7%	5.6 ± 1.6%	20.6 ± 2.3%	11.8 ± 3.0%
P0 (*n* = 3 brains)	P1–P5	0%	0%	0%	1.1 ± 2.0%

### Embryonic Proliferation of GAD67^+^ Intermediate Progenitors of GABAergic Inhibitory Neurons (IPGNs)

**FIGURE 4:**
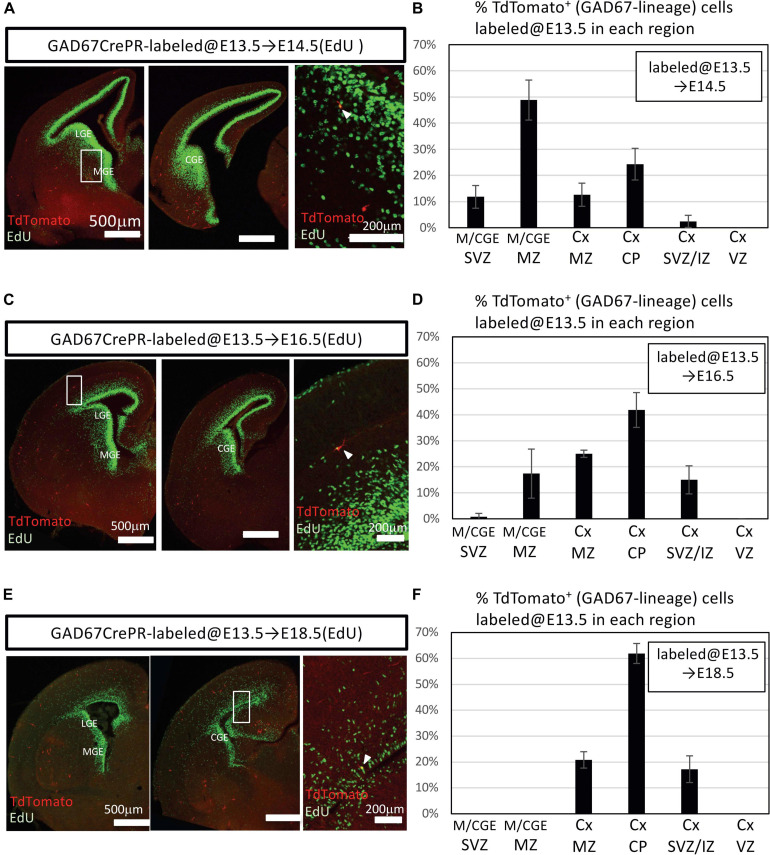
Embryonic proliferation of GAD67-lineage cells. **(A)** Coronal section of a GAD67-CrePR;Ai9 mouse brain at E14.5 with EdU staining (green). Mifepristone was administered to pregnant GAD67-CrePR;Ai9 mice at E13.5, and EdU was injected i.p. 30 min before fixation. Middle image shows higher magnification of region outlined with white boxes in left column. White arrowhead indicates a double-positive cell in SVZ of MGE. Ctx, cortex; MGE, medial ganglionic eminence; LGE, lateral ganglionic eminence; CGE, caudal ganglionic eminence. **(B)** Percentages of TdTomato^+^ cells (GAD67 lineage labeled at E13.5) labeled with EdU (E14.5) in each region. Error bars represent SDs (*n* = 3 brains). Ctx, cortex; M/CGE, medial/caudal ganglionic eminence; MntZ, mantle zone; MrgZ, marginal zone; CP, cortical plate; SVZ/IZ, subventricular zone/intermediate zone; VZ, ventricular zone. **(C)** Coronal section of a GAD67-CrePR;Ai9 mouse at E16.5 with EdU staining (green). Mifepristone was administered to pregnant GAD67-CrePR;Ai9 mice at E13.5, and EdU was injected i.p. 30 min before fixation. Middle image shows higher magnification of region outlined with white box. White arrowhead indicates a double-positive cell in cortical plate. **(D)** Percentages of TdTomato^+^ cells (GAD67 lineage labeled at E13.5) labeled with EdU (E16.5) in each region. Error bars represent SDs (n = 3 brains). **(E)** Coronal section of a GAD67-CrePR;Ai9 mouse brain at E18.5 with EdU staining (green). Mifepristone was administered to pregnant GAD67-CrePR;Ai9 mice at E13.5, and EdU was injected i.p. 30 min before fixation. White arrowhead indicates a double-positive cell in SVZ/IZ. **(F)** Percentages of TdTomato^+^ cells (GAD67 lineage labeled at E13.5) labeled with EdU (E18.5) in each region. Error bars represent SDs (*n* = 3 brains).

Our data revealed a small number of postnatally proliferative IPGNs (Figures 2, 3). Moreover, the cortical GABAergic neurons that derived from postnatally proliferative IPGNs survived into later postnatal development. On the other hand, significantly more GABAergic neurons that were derived from the GABAergic neuron progenitors were produced during embryonic stages. However, the proportion of the GABAergic neurons derived from the IPGNs in each embryonic stage was unclear. To define the distribution patterns of embryonically proliferating IPGNs, we analyzed E14.5, E16.5, and E18.5 GAD67-CrePR;Ai9 mice that were administered mifepristone at E13.5 to label GAD67^+^ cells, which were sacrificed 30 min after EdU injection (Figure 4). At E14.5, 3.5 ± 0.4% of the TdTomato^+^ cells were EdU^+^ (27/793 cells from three brains). At E16.5, 0.6 ± 0.3% of the TdTomato^+^ cells were EdU^+^ (4/672 cells from three brains). At E18.5, 0.8 ± 0.2% of the TdTomato^+^ cells were EdU^+^ (5/584 cells from three brains). We found that approximately half of the GAD67-lineage TdTomato^+^ cells migrated to the cortex and the other half were in the M/CGE-mantle zone and M/CGE-SVZ at E14.5 (Figures 4A,B). These cells migrated tangentially toward the cerebral cortex via two major migratory streams, a superficial route through the cortical-marginal zone and a deeper route through the cortical-SVZ/IZ, consistent with previous reports (Lavdas et al., 1999; Wichterle et al., 2001; Tanaka et al., 2006, 2009; Miyoshi and Fishell, 2011; Lim et al., 2018). Furthermore, 3.5% of EdU^+^ GAD67-lineage TdTomato^+^ double-positive cells were observed in the MGE-SVZ/IZ or M/CGE-mantle zone at E14.5 (Figures 4A,B). These results suggest that ∼3.5% of the embryonic proliferating IPGNs mainly populated the M/CGE-SVZ/IZ or M/CGE-mantle zone at E14.5. However, no GAD67-lineage TdTomato^+^ cells were found in the M/CGE at E18.5. Therefore, migration of the E13.5-labeled GAD67-lineage-TdTomato^+^ cells from the M/CGE to the cerebral cortex had already finished by E18.5. In addition, a very small proportion (<1%) of the GAD67-lineage TdTomato^+^ cells colabeled with EdU in the cortex in the cortical plate and cortical-SVZ/IZ at E16.5 and E18.5 (Figures 4C–F). These results suggest that embryonic brains have multiple regions for embryonic IPGN proliferation. Furthermore, a substantial proportion of the GAD67^+^ IPGNs actively proliferate at around E14.5, and their proliferative activities decline with tangential migration to the cortex.

### A Large Proportion of GAD67^+^ Embryonic IPGNs Proliferate and Survive in Cerebral Cortex During Development

**FIGURE 5:**
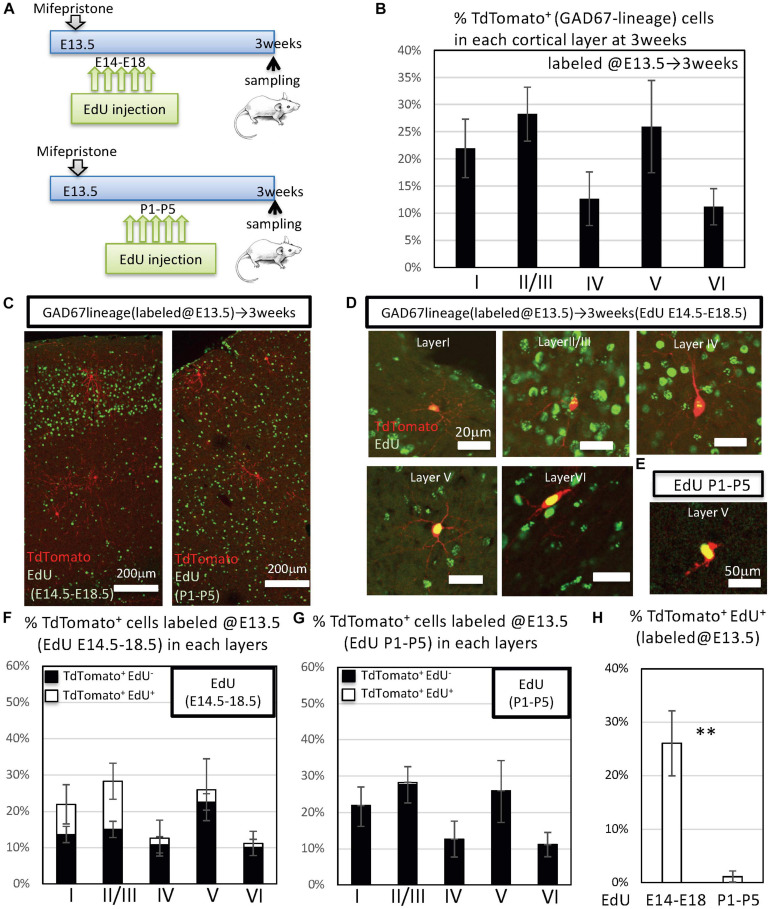
Fate mapping analysis of GAD67^+^ IPGNs in GAD67-CrePR mice labeled at E13.5. **(A)** Experimental design for labeling IPGNs at the embryonic stage. Mifepristone was administered to pregnant GAD67-CrePR;Ai9 mice at E13.5, and EdU was injected i.p. from E14.5 to E18.5 or P1 to P5. Mifepristone- and EdU-treated mice were analyzed at 3 weeks. **(B)** Percentages of TdTomato^+^ GAD67-lineage cells labeled at E13.5 in each cortical layer. Error bars represent SDs (*n* = 9 brains). **(C)** Coronal section of GAD67-CrePR;Ai9 mouse labeled at E13.5 at 3 weeks with EdU staining (green). EdU was injected E14.5–E18.5 (left) P1–P5 (right) by dual-fluorescence immunohistochemistry. **(D)** Representative images of TdTomato^+^ GAD67-lineage cells labeled at E13.5 with EdU (E14–18) (green) in each cortical layer at 3 weeks. **(E)** Representative image of TdTomato^+^ GAD67-lineage cells with EdU (P1–P5) (green) in cortical layer V at 3 weeks. **(F)** Percentages of TdTomato^+^ cells labeled at E13.5 with EdU (E14–E18) in each cortical layer. Error bars represent SDs (*n* = 6 brains). **(G)** Percentages of TdTomato^+^ cells labeled at E13.5 with EdU (P1–P5) in each cortical layer. Error bars represent SDs (*n* = 6 brains). **(H)** Percentages of TdTomato^+^ EdU^+^ GAD67-lineage cells labeled at P0 and EdU injected from E14.5 to E18.5 (*n* = 6 brains) or P1 to P5 (*n* = 3 brains). ^∗∗^*P* < 0.01. Error bars represent SDs.

To determine the ratio of embryonically to postnatally proliferative IPGNs, mifepristone was administered to pregnant GAD67-CrePR;Ai9 mice at E13.5 followed by EdU injections from E14.5 to E18.5 or from P1 to P5 to label proliferative cells (Figure 5A). At 3 weeks after birth, the TdTomato^+^ cells labeled by mifepristone administration at E13.5 were broadly distributed throughout the brain, including in the cerebral cortex (Figures 5B–D and Supplementary Figure 3). Interestingly, the distribution pattern of the TdTomato^+^ cells labeled at E13.5 was distinct from that observed when mifepristone was injected at P0 (Figures 2B, 5B). The TdTomato^+^ cells labeled at E13.5 were observed throughout the cortical layers and tended to accumulate in each cortical layer (Figures 5B–D). To assess the ratio of the TdTomato^+^ GAD67-lineage EdU^+^ double-positive cells in each layer, we performed immunohistochemical analyses. The TdTomato^+^ GAD67-lineage cells and EdU^+^ cells were detected throughout all cortical layers by immunohistochemistry (Figures 5C,D). The embryonically labeled EdU^+^ cells (E14–E18) accumulated in the upper layers (i.e., layers II/III), whereas the postnatally labeled EdU^+^ cells (P1–P5) were scattered in each cortical layer.

Approximately 26.1% [±6.1% (292/1,130 cells from six brains)] (Figures 5F–H) of the TdTomato^+^ GAD67-lineage cells (mifepristone administration at E13.5) colabeled with EdU injected from E14.5 to E18.5. By contrast, 1.1 ± 1.1% (6/495 cells from three brains) (Figures 5F–H) of the TdTomato-positive GAD67-lineage cells (mifepristone administration at E13.5) colabeled with EdU injected from P1 to P5 (Figure 5G). Thus, the number of embryonically proliferative IPGNs (EdU injection at E14.5–E18.5) was nearly 24-fold higher than that of the postnatally proliferative IPGNs (EdU injection at P1–P5), suggesting that significantly more IPGNs are produced during embryonic stages. These embryonically proliferating cells tended to localize to the superficial layers (i.e., layers II/III) rather than the deep cortical layers at 3 weeks after birth in comparison to the GAD67-lineage cells labeled at P0 (Figures 2F,G, 5F,G and Table 2). By contrast, a very small number of postnatally proliferative IPGNs (mifepristone administration at E13.5 and EdU injection at P1–P5) was observed in the cerebral cortex 3 weeks after birth in our fate mapping study. These results delineate the fate of late embryonic and perinatal proliferative IPGNs.

### Subtype Differentiation (Specification) of Proliferative GAD67-Lineage Cells Derived From Embryonic Intermediate Progenitors of GABAergic Inhibitory Neurons (IPGNs)

**FIGURE 6:**
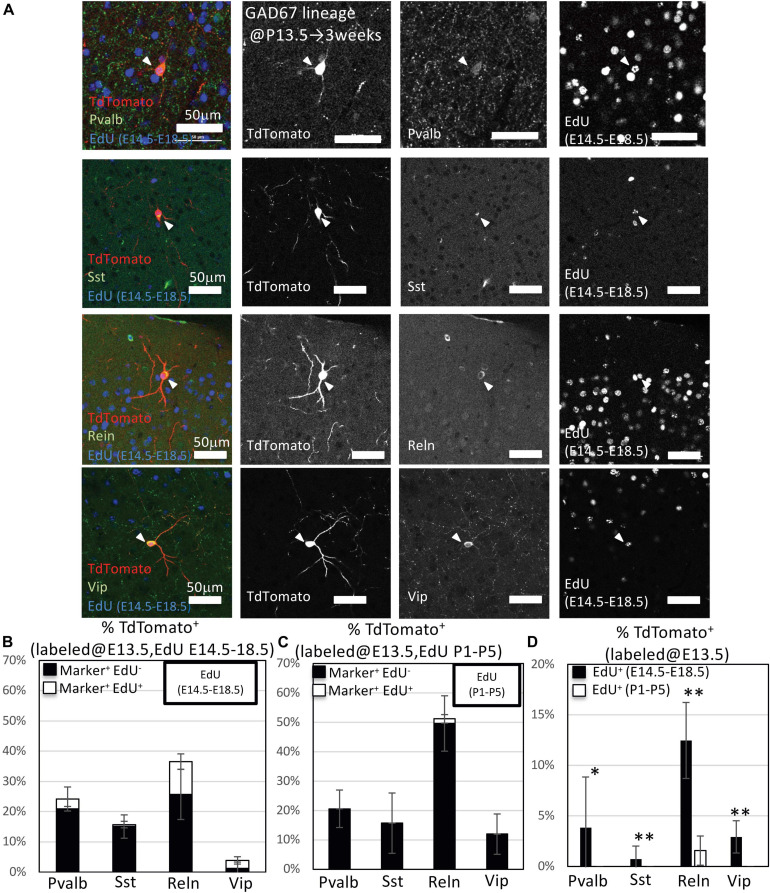
Immunohistochemistry analysis of GAD67-lineage cells labeled at E13.5. **(A)** Triple-fluorescence immunohistochemistry of GAD67-lineage cells (red; labeled at E13.5) for EdU (green; labeled E14.5–E18.5) and GABAergic (Pvalb, Sst, Reln, and Vip) neuron markers (green) from GAD67-CrePR;Ai9 mice at 3 weeks of age. White arrowheads indicate the triple-positive cells. Pvalb, parvalbumin; Sst, somatostatin; Reln, reelin; Vip, vasoactive intestinal peptide. **(B)** Percentage of TdTomato^+^ cells labeled with EdU (E14–E18), and/or GABAergic (Pvalb, Sst, Reln, and Vip) neuron subtype markers. Black bars indicate the percentages of TdTomato^+^ and GABAergic neuron subtype marker double-positive cells. White bars indicate the percentages of TdTomato^+^ GABAergic neuron subtype marker and EdU^+^ triple-positive cells. **(C)** Percentage of TdTomato^+^ cells labeled with EdU (P1–P5) and/or GABAergic (Pvalb, Sst, Reln, and Vip) neuron subtype markers. Black bars indicate the percentages of TdTomato^+^ and GABAergic neuron subtype marker double-positive cells. White bar indicates the percentage of TdTomato^+^ GABAergic neuron subtype marker and EdU^+^ triple-positive cells. **(D)** Percentages of TdTomato^+^ EdU^+^ GABAergic neuron subtype marker^+^ GAD67-lineage cells labeled at E13.5 and EdU injected from E14.5 to E18.5 or P1 to P5. ^∗^*P* < 0.05; ^∗∗^*P* < 0.01.

We next performed immunohistochemistry to examine the ratios of Pvalb^+^, Sst^+^, Reln^+^, and Vip^+^ GABAergic neurons derived from the embryonic IPGNs in GAD67-CrePR;Ai9 mice administered mifepristone at E13.5. At 3 weeks, almost all of the cortical TdTomato^+^ cells colabeled with NeuN, a marker for mature neurons, and >97% of the cells were positive for gamma-aminobutyric acid (GABA) (Supplementary Figure 4). To investigate subtypes of cortical GABAergic neurons which were produced from embryonic GAD67-lineage IPGNs during late embryonic stages (EdU injection at E14.5–E18.5) or perinatal stages (EdU injection at P1–P5), we performed immunohistochemical analyses of Pvalb, Sst, Reln, and Vip with EdU staining in the GAD67-CrePR;Ai9 mice administered mifepristone at E13.5 to label GAD67^+^ cells (Figure 6A). Among the TdTomato^+^ GAD67-lineage cells, 22.7 ± 4.3% were Pvalb^+^ (71/312 cells from six brains), 15.9 ± 8.7% were Sst^+^ (50/315 cells from six brains), 42.6 ± 8.9% were Reln^+^ (135/316 cells from six brains), and 10.4 ± 8.6% were Vip^+^ (34/321 cells from six brains) (Figure 6B and Table 3). These results suggest that GAD67-lineage cells that were labeled at E13.5 differentiated into Pvalb^+^, Sst^+^, Reln^+^, and Vip^+^ GABAergic neurons. Interestingly, nearly half of the TdTomato^+^ GAD67-lineage cells were Reln^+^. We then estimated the ratio of each GABAergic neuron subtype marker and EdU double-positive cells among the TdTomato^+^ GAD67-lineage cells (EdU injections from E14.5 to E18.5 or from P1 to P5). Among the TdTomato^+^ GAD67-lineage cells that were labeled with EdU injected at E14.5–E18.5, 3.2 ± 4.0% (5/153 cells from brains) were double positive for Pvalb and EdU, 0.6 ± 1.1% (1/153 cells from three brains) were double positive for Sst and EdU, 10.8 ± 2.6% (17/156 cells from three brains) were double positive for Reln and EdU, and 2.6 ± 1.2% (4/156 cells from three brains) were double positive for Vip and EdU (Figure 6D and Table 3). Thus, the GAD67-positive IPGNs that proliferated during the late embryonic stages differentiated into Pvalb^+^, Sst^+^, Reln^+^, and Vip^+^ GABAergic cortical neurons derived from MGE and CGE. In addition, these embryonically proliferative IPGNs preferentially produced Reln^+^ cortical GABAergic neurons, which may be equivalent to CGE-derived GABAergic neurons (Miyoshi and Fishell, 2011). No double-positive cells were found for Pvalb (0/159 cells from three brains), Sst (0/156 cells from three brains), or Vip (0/156 from brains) among the TdTomato^+^ GAD67-lineage cells labeled by EdU injection at postnatal stages (P1–P5), whereas only 1.6 ± 1.4% (3/160 cells from three brains) were double positive for Reln and EdU (Figures 6C,D). The proportion of the GAD67-positive IPGNs that proliferated during the late embryonic stage (E14.5–E18.5) was significantly higher than that during the perinatal stage (*P* < 0.01) for each GABAergic neuron subtype. These results demonstrate that only a very small number of IPGNs postnatally proliferate in the cerebral cortex and that these cells are derived from the CGE.

## Discussion

Cortical GABAergic interneurons comprise approximately 20% of all neurons (Tamamaki et al., 2003; Sahara et al., 2012) and are generated from apical neural progenitors as well as intermediate progenitors of IPGNs (Wu et al., 2011). The appropriate distribution of GABAergic neurons throughout the neocortex requires not only migratory guidance but also proper regulation of progenitor proliferation. Here, we characterized the spatiotemporal distribution of IPGN-derived cortical GABAergic neurons by using GAD67-CrePR mice, thereby providing basic and fundamental information toward understanding the mechanisms regulating the development of cortical GABAergic neurons.

Glutamic acid decarboxylase 67 (encoded by *Gad1*) and GAD65 (encoded by *Gad2*) are enzymes that catalyze the production of GABA from glutamic acid. While GAD67 and GAD65 are expressed in mature GABAergic neurons, a small portion of the GAD67/65^+^ cells in the embryonic (M/C) GE and perinatal cortex in rodents and monkeys express proliferative makers (Inta et al., 2008; Wu et al., 2011; Plachez and Puche, 2012; Riccio et al., 2012). These observations indicate that GAD67 and GAD65 are expressed in IPGNs as well as mature GABAergic neurons in rodents and monkeys. Consistent with this, Dlx2, a transcription factor expressed by IPGNs (Hansen et al., 2013; Petros et al., 2015), binds to the promoter regions of *Gad1* and *Gad2* (Le et al., 2017). Furthermore, gene expression profiling, single-cell RNA-sequencing, and microarray analyses using cortical GABAergic neurons and/or their progenitors have revealed that GAD67^+^ cells can transition from apical progenitors to differentiated mature cortical GABAergic neurons (Esumi et al., 2008; Mayer et al., 2018; Mi et al., 2018). Thus, proliferative GAD67^+^ cells should be considered late-state intermediate/basal progenitors of cortical GABAergic neurons.

Our fate mapping analyses revealed that the GAD67-lineage cells labeled at P0 tended to distribute to the superficial cortical layers compared to those labeled at E13.5 (Figure 2B). Approximately 70% of cortical GABAergic neurons reportedly derive from the MGE, whereas ∼30% derive from the CGE, and early-born GABAergic neurons tend to locate to the deeper layers of rodent cortex, whereas late-born GABAergic neurons are found in superficial layers (Miller, 1985; Fairén et al., 1986; Rymar and Sadikot, 2007; Bartolini et al., 2013).

Cortical GABAergic neurons can be classified as Pvalb^+^, Sst^+^, Reln^+^, and Vip^+^ (Miyoshi et al., 2010; Brandão and Romcy-Pereira, 2015). Whereas Sst^+^ neurons are produced during the early stage of neurogenesis, a large proportion of the Pvalb^+^ neurons are generated in the MGE during the late stages (Rymar and Sadikot, 2007). On the other hand, the CGE-derived GABAergic neurons are produced in late embryonic stages and mainly express either Reln or Vip (Nery et al., 2002; Miyoshi et al., 2010; Rudy et al., 2011; Vitalis and Rossier, 2011). Reln^+^ neurons are derived from both the CGE and MGE, as subpopulations of the MGE-derived Sst^+^ neurons coexpress Reln (Miyoshi et al., 2010; Miyoshi and Fishell, 2011). Recently, a number of the CGE-derived GABAergic neurons, which express Reln and Vip, were found to populate superficial cortical layers independently of their birthdate (Miyoshi et al., 2010), and their peak production was during late embryonic stages (Miyoshi and Fishell, 2011). In primates, the IPGNs (basal/non-epithelial progenitors) derived from CGE contribute to a large number of cortical GABAergic neurons (Hansen et al., 2013). These previous reports indicate that the specification of the GABAergic neuron subtypes depends on their birthdates and localization.

Our data indicated that embryonically proliferative GAD67-lineage cells (labeled at E13.5) tend to populate the superficial cortical layers. From the view of proliferative IPGNs (labeled at E13.5), approximately 63% expressed Reln, and ∼15% of those were Vip, but only <1% were labeled Sst (Figure 6F). Our results indicate that more than half of cortical IPGNs during late embryonic stage are derived from the CGE. This tendency may correspond to primate cortical GABAergic neuron development.

Several lines of evidence indicate that GABAergic neuron progenitors in the MGE produce both Pvalb^+^ and Sst^+^ cortical GABAergic neurons (Brown et al., 2011; Ciceri et al., 2013; Harwell et al., 2015; Mayer et al., 2015). Sst^+^ GABAergic neurons are principally produced from early-stage GABAergic neuron progenitors (∼E12.5), whereas Pvalb^+^ GABAergic neurons are continuously produced throughout development (Miyoshi et al., 2007; Wonders et al., 2008; Inan et al., 2012). However, these reports did not distinguish apical (epithelial) progenitors that divide in the ventricular zone (VZ) from IPGNs (basal/non-epithelial progenitors) that divide in the SVZ (Pilz et al., 2013). Basal/non-epithelial progenitors in the SVZ of the MGE mainly produce Pvalb^+^ rather than Sst^+^ cortical GABAergic neurons (Petros et al., 2015). Accordingly, our immunohistochemical analyses showed that GAD67^+^ IPGNs that derive from MGE (Pvalb^+^ or Sst^+^) and travel along the migratory stream to the cortex in late embryonic stages (E14–E18) tend to become Pvalb^+^ neurons, although a small proportion become Sst^+^ neurons. Thus, GAD67^+^ IPGNs in embryonic stages derived from the MGE are most likely to have and retain characteristics of late-stage basal progenitors.

Cortical GABAergic neurons are produced from cortical layer 1 GABAergic neuron progenitor cells (L1-INP cells) (Ohira et al., 2010), and the generation of L1-INP cells increases under ischemic conditions and after treatment with fluoxetine, a selective serotonin reuptake inhibitor (Ohira et al., 2013). We observed that a small number of postnatal proliferative GAD67^+^ IPGNs that were labeled after mifepristone administration at E13.5 and P0 survived throughout postnatal development and tended to populate the superficial cortical layers (Figures 2G, 5G). These results suggest that GAD67^+^ IPGNs are maintained in the cortex and may act as L1-INP cells in adult brain.

In conclusion, the present study demonstrates that GAD67^+^ IPGNs, which are considered late-stage basal progenitors of cortical GABAergic neurons, substantially contribute to the GABAergic neuron population in adult cerebral cortex. In addition, the proliferative rates of IPGNs and the laminar distributions of their progenies change during cortical development. Thus, the characteristic features of IPGNs are spatiotemporally regulated during brain development.

## Data Availability Statement

The original contributions presented in the study are included in the article/Supplementary Material, further inquiries can be directed to the corresponding author/s.

## Ethics Statement

The animal study was reviewed and approved by the rules for animal care and use for research and education of Kumamoto University.

## Author Contributions

All authors listed have made a substantial, direct and intellectual contribution to the work, and approved it for publication. SE, NT, and TF coordinated the project. KS generated GAD67-CrePR mice. TS and YY provided transgenic mice. SE conceived and designed the study and performed experiments, collected data, and co-wrote the manuscript with TK, MN, KoM, KeM, TS and YY helped to perform the experiments. All authors have seen and agreed with the content of the manuscript.

## Conflict of Interest

The authors declare that the research was conducted in the absence of any commercial or financial relationships that could be construed as a potential conflict of interest.
